# In vivo endothelial gene regulation in diabetes

**DOI:** 10.1186/1475-2840-7-8

**Published:** 2008-04-19

**Authors:** J Gregory Maresh, Ralph V Shohet

**Affiliations:** 1 Department of Medicine, University of Hawaii John A. Burns School of Medicine, Honolulu, USA

## Abstract

**Background:**

An authentic survey of the transcript-level response of the diabetic endothelium in vivo is key to understanding diabetic cardiovascular complications such as accelerated atherosclerosis and endothelial dysfunction.

**Methods:**

We used streptozotocin to induce a model of type I diabetes in transgenic mice that express green fluorescent protein under the control of an endothelial-specific promoter (Tie2-GFP) allowing rapid isolation of aortic endothelium. Three weeks after treatment, endothelial cells were isolated from animals with blood glucose > 350 mg/dl. Aortae from the root to the renal bifurcation were rapidly processed by mincing and proteolytic digestion followed by fluorescent activated cell sorting to yield endothelial cell populations of >95% purity. RNA was isolated from >50,000 endothelial cells and subjected to oligo dT amplification prior to transcriptional analysis on microarrays displaying long oligonucleotides representing 32,000 murine transcripts. Five regulated transcripts were selected for analysis by real-time PCR.

**Results:**

Within replicate microarray experiments, 19 transcripts were apparently dysregulated by at least 70% within diabetic mice. Up-regulation of glycam1, slc36a2, ces3, adipsin and adiponectin was confirmed by real-time PCR.

**Conclusion:**

By comprehensively examining cellular gene responses in vivo in a whole animal model of type I diabetes, we have identified novel regulation of key endothelial transcripts that likely contribute to the metabolic and pro-inflammatory responses that accompany diabetes.

## Background

Diabetes increases mortality and morbidity in large part due to cardiovascular events [1]. Vascular changes associated with diabetes include endothelial damage and dysfunction [2] that contribute to accelerated atherosclerosis and the development of hypertension [3]. Diabetic endothelial damage is likely multifactorial, involving numerous stresses that impinge on the endothelial cells in vivo. These include the generation of advanced glycation end-products [4], the effects of dyslipidemia [5], the generation of reactive oxygen and nitrogen species [6], and altered insulin signaling [7]. Many of the chronic endothelial effects of diabetes will be reflected in transcriptional regulation, either as a direct response to hyperglycemia and abnormalities of insulin signaling, or as a secondary response to the effects of these stresses. An assessment of the underlying in vivo transcriptional changes associated with the earliest stages of insulin deficiency will enhance our understanding of the endothelial response in diabetes, and suggest pathways and candidate genes that contribute to endothelial dysfunction and vascular disease.

Previous in vitro studies of the transcriptional response of cultured endothelial cells to hyperglycemia have been performed using macroarrays [8]. However, such an analysis is not likely to capture the integrated organismal response, which will include systemic effects of altered energy metabolism, interaction among different cell types in the vessel wall, and other global effects of altered insulin signaling. By comprehensively examining endothelial gene regulation in vivo in the diabetic animal, we expect to derive a more robust and authentic view of the components of pathways that are responsible for accelerated atherogenesis, the most important cause of diabetic morbidity and mortality.

## Methods

### Animals

Mice homozygous for the Tie2-GFP transgene (Tg [TIE2GFP]287Sato, stock number 003658) were obtained from Jackson labs (Bar Harbor, ME) and bred for these experiments. Males were used at 6–10 weeks of age. Controls were siblings of the treated animals. All procedures were approved by the Institutional Animal Care and Use Committee of the University of Hawaii.

### Induction of diabetes

Following an overnight fast, a single 180 mg/kg dose of streptozotocin (STZ, Sigma, St. Louis, MO) was injected into the peritoneum. Control animals received injections of sterile saline. Animals were allowed to feed ad libitum on standard lab diet. 21 days after receiving injections, glucose levels in cardiac blood were assessed by glucometer (Accucheck, Becton Dickinson). STZ-injected animals (with a glucose level >350 mg/dL) and saline-injected animals (with glucose <200 mg/dL) were processed on the same day. In each of two independent experiments, pooled cells from four experimental vs. four control mice were collected 21 days after receiving injections.

### Cell Isolation

Animals were euthanized by CO_2 _asphyxiation. The aortae from the iliac bifurcation to the aortic root were excised by dissection and freed of adherent tissue. The luminal blood was removed and the aortas were sliced into 1 mm segments. The aortic segments pooled from 4 animals were suspended in 5 ml of Dulbecco's PBS with dextrose 2 mg/ml. The suspension was combined with 5 ml of prewarmed PBS containing 10 mg/ml type II collagenase (Worthington), and 60 units/ml deoxyribonuclease I. The suspension was agitated continuously at 37°C on a shaking platform, and triturated 10 times every 10 minutes for a total digestion period of 40 min to generate a single cell suspension. The cell suspension was maintained at 0–4°C throughout the remainder of the isolation, which lasted 2–3 hours total. The suspension was combined with 10 ml of 10% fetal bovine serum (FBS) in Dulbecco's Modified Eagle's Medium and cells were collected by centrifugation and resuspended in 10 ml of PBS. This suspension was then filtered through a sterile 40 um mesh filter to remove undigested tissue fragments. Following centrifugation, the pellet was resuspended in 0.3 ml PBS containing 0.5 mM EDTA, 30 U/ml deoxyribonuclease I, 3% FBS, and 2 mg/ml dextrose. The suspension was once again filtered through a 40 um mesh filter.

The resulting aortic cell suspensions were sorted using a MoFlo from Dako Cytomation (Carpinteria, CA) or a FacsAria from Becton Dickinson. Cells were excited by a 488 nm laser and GFP signals collected at 510 to 550 nm. Positive cells were collected directly into Trizol (Invitrogen, Carlsbad CA) and processed according to the manufacturer's protocol. Glycogen was added to facilitate precipitation of the sub-microgram quantities of RNA thus obtained.

In addition to sorted endothelium, whole aortae from 4 diabetic and 4 control mice were individually collected 21 days after injections, rapidly frozen, extracted with Trizol, and analyzed by real-time PCR.

RNA was purified further using the Micro RNEasy system (Qiagen) according to the manufacturer's protocol. The Qubit system (Invitrogen, Carlsbad CA) was used to fluorometrically quantify the resultant RNA. Up to 20 ng RNA isolated from cells was then subjected to two cycles of amplification [9]. This produced 5–30 ug of cRNA for use in microarray analysis and real-time PCR. Endothelial RNA was also amplified with the SPIA system (Nugen, San Carlos, CA) according to the manufacturer's protocol and the resultant cDNA was analyzed by real-time PCR.

### Microarray analysis

2 ug of cRNA from diabetic and control aortic cells were labeled directly with Cy3 and Cy5 fluorophores with the MicroMax labeling kit (Perkin Elmer, Waltham, MA) according to the manufacturer's instructions and hybridized overnight to microscope slides containing the Operon V3 long oligo array. Stringency washing at room temperature was sequentially performed for ten minutes in each of the following solutions: 2× SSC containing 0.1% SDS, 0.1× SSC containing 0.1% SDS, and finally, 0.1× SSC. Slides were scanned using the Genepix 4000B^® ^(Molecular Devices, Union City, CA). Replicate hybridizations were performed (with Cy3 and Cy5 dye-swapped) and were analyzed with Genepix^® ^and Acuity^® ^software from Molecular Devices.

The statistical analysis of microarray results was performed using the functions available within Acuity^®^. Initial results were normalized using the ratio of medians method and filtered to exclude those with any of the following characteristics: a percentage of saturated pixils >3, a signal/noise ratio <3, a (regression ratio 635/532)^2 ^< 0.6, or a Genepix flag. Filtered results exhibiting up-regulation (a log_2 _[fold change] >0.75) or down-regulation (log_2 _[fold change] <0.75) were tabulated. (A log_2 _[fold change] of 0.75 corresponds to 1.7-fold on the linear scale). The one sample t-test function in Acuity was applied to the tabulated results.

### Real-time PCR confirmation

Primer sequences are listed in Table 1. Superscript II (Invitrogen) was used according to the manufacturer's instructions to generate cDNA from cRNA. All primer sets were assessed by gel electrophoresis to confirm amplification from endothelial cDNA of a single band of the expected size. Primers were used to amplify product from cDNA representing 5 ng of cRNA. PCR was run in triplicate with SYBR^® ^green fluorophore (Molecular Probes, Portland, OR) in an Opticon™ device (MJ Research, Waltham, MA). A standard two-phase reaction (95°C 15 sec, 60°C 1 min) worked for all amplifications.

**Table 1:** Real-time PCR primer sequences

Transcript	RefSeq ID	Forward	Reverse
Adiponectin	NM_009605	TCAGTGGATCTGACGA CACCA	AGCTTGCAACAGTAG CATCCTG
Ces3	NM_053200	AGAGCCCTGGAGCTTC GTG	GAGCACATAGGCGGG TAGGAG
Adipsin	NM_013459	GCAGTGGGTGCTCAGT GCT	TCGTCATCCGTCACTC CATC
Slc36a2	NM_153170	CCGCTCTTCTTTGGAAC AGC	GACCACACCGATGCTT TCAA
Glycam1	NM_008134	CACAGATGCCATTCCAG CTG	TCACTGGTGTAGCTGG TGGG

The relative expression level for each gene was interpolated from a standard curve generated from a series of cDNA dilutions at cycle times where C_t_, the threshold intensity, was clearly exceeded. In each real-time PCR run, the abundance of GAPDH was assessed in parallel, and expression values for genes of interest were calculated by normalization to GAPDH as a loading control. Fold changes represent the ratio of diabetic to control expression values. The statistical analysis of real-time PCR results was performed using the t-test within Microsoft Excel^® ^to compare diabetic vs. control expression values.

## Results

**Figure 1:**
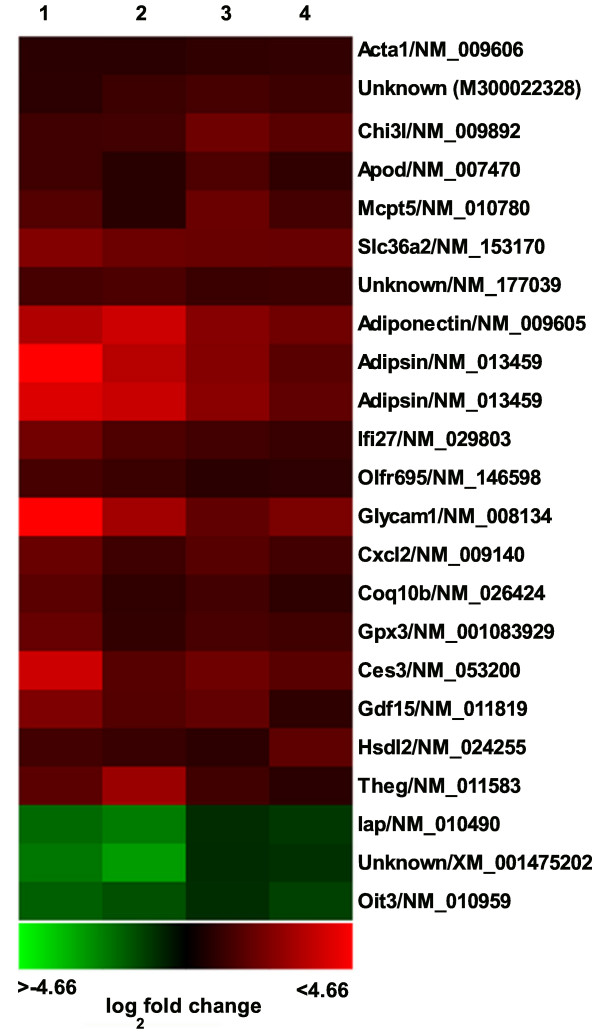
**Changes in mRNA abundance 21 days after streptozotocin exposure as determined by microarray analysis**. This heat map shows 23 features found consistently dysregulated by greater than 1.7-fold in diabetic vs. control endothelium. Green indicates down-regulation and red indicates up-regulation. Each column represents a single microarray analysis. Columns 1–2 and columns 3–4 display the results of separate experiments. Column 2 and 4 represent dye-reversed arrays of 1, and 3, respectively.

**Figure 2:**
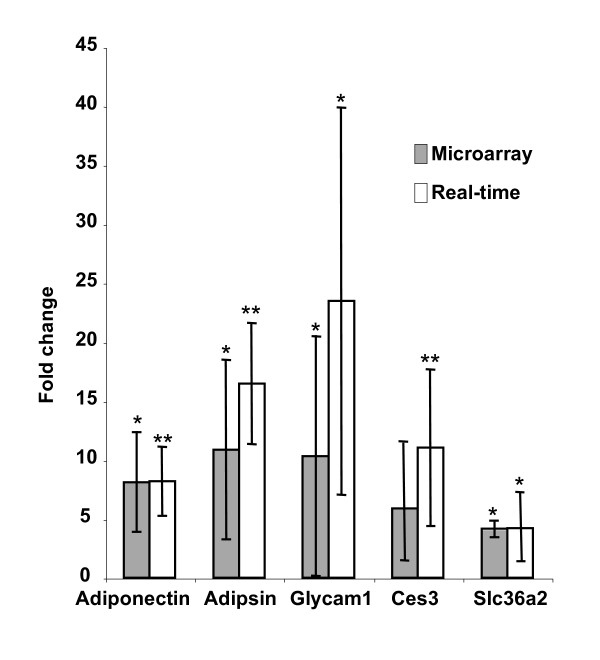
**Comparison of real-time PCR and microarray results**. Combined real-time PCR and microarray results of selected genes uncovered by the microarray analysis described in table 1. * indicates a p-value < .05, ** indicates a p-value < .005.

Microarray results have been placed in the GEO database under series record GSE9072. Transcripts within the aortic endothelium dysregulated by at least 70% in response to the type I diabetic model are shown in fig. 1. The average microarray-based fold change, reference sequence identity, and p-value are included in Table 2. Five transcripts displaying a high level of dysregulation in replicate array hybridizations were chosen for analysis by real-time PCR. As shown in fig. 2, there is concordance between the measurement of the diabetic response by microarray vs. real-time PCR.

**Table 2:** Average expression fold change in diabetic vs. control endothelium

Name	Operon ID	RefSeq ID	Fold Change	P-value
ApoD (apolipoprotein D)	M200000796	NM_007470	2.1	0.02
Adiponectin	M200001531	NM_009605	8.1	0.01
Cxcl2	M200002070	NM_009140	2.8	0.19
Glutathione peroxidase 3	M200002493	NM_001083929	2.6	0.28
Hsdl2 (hydroxysteroid dehydrogenase-like 2)	M200005344	NM_024255	2.3	0.01
Coq10b (coenzyme Q10 homolog B)	M200005601	NM_026424	2.3	0.18
Gdf15 (growth/differentiation factor 15)	M200007081	NM_011819	3.3	0.06
Acta1 (alpha actin, skeletal muscle)	M200013335	NM_009606	1.8	0.08
Oit3 (oncoprotein-induced transcript 3)	M300001065	NM_010959	0.4	0.13
Adipsin	M300002011	NM_013459	10.9	0.03
Adipsin	M300002012	NM_013459	9.5	0.03
Slc36a2	M300002100	NM_153170	4.1	0.02
Theg (testicular haploid expressed gene)	M300002133	NM_011583	3.6	0.01
Ifi27 (interferon alpha-inducible protein 27)	M300002619	NM_029803	2.8	0.05
Mcpt5 (mast cell chymase)	M300003152	NM_010780	2.7	0.13
Glycam1	M300003316	NM_008134	10.3	0.02
Ces3 (carboxylesterase 3)	M300007868	NM_053200	5.9	0.08
Chi3l (chitinase 3-like 3 or -4)	M300012867	NM_009892 NM_145126	2.9	0.01
Unknown	M300014207	NM_177039	2.3	0.08
Iap (IgE binding protein)	M300014963	NM_010490	0.4	0.11
Olfr695 (olfactory receptor 695)	M300017077	NM_146598	2.0	0.20
Unknown	M300019951	XM_001475202	0.4	0.08
Unknown	M300022328	--	2.1	0.06

Enzymatically isolated preparations of adipose stromal cells exhibiting contamination by small immature adipocytes has been reported [10]. In our studies, we consider contamination of the sorted endothelium product by small adipose cells to be unlikely, due to the aortic source tissue and high degree of purification achieved by FACS. Moreover, in results not shown, SVEC cells, an SV-40 immortalized line of endothelial cells, do indeed express adiponectin as determined by real-time PCR.

**Figure 3:**
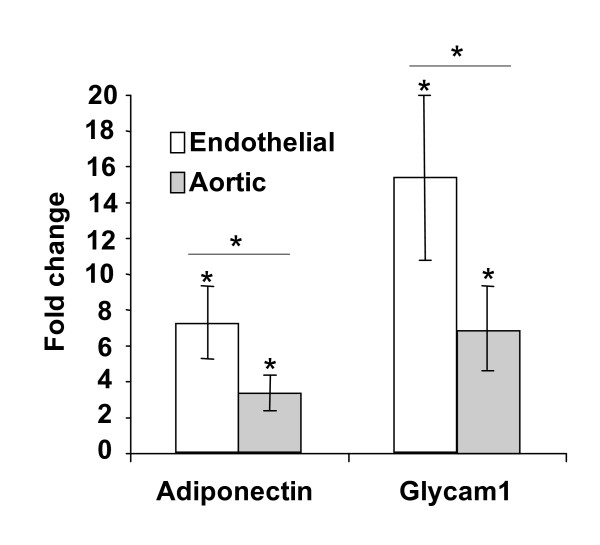
**Adiponectin and glycam 1 expression in whole aorta vs. aortic endothelium**. Diabetes-induced changes in mRNA abundance of adiponectin and glycam 1 in whole aorta vs. aortic endothelium, measured by real-time PCR. * indicates a p-value < .05.

Dysregulation of glycam1 and adiponectin was confirmed in a third independent experiment in which an alternative amplification system, the SPIA system, was employed to amplify RNA directly into cDNA. This endothelial regulation, shown in fig. 3, was determined using real-time PCR and is in agreement with the microarray and real-time analysis shown in fig. 2. Also shown in fig. 3, a similar analysis of whole aortic tissue from diabetic mice reveals much less dysregulation. The smaller response of Glycam 1 and adiponectin within diabetic aorta vs. the FACS-sorted product most likely reflects the relatively low proportion of endothelial cells in whole aorta.

## Discussion

In our survey of the endothelial response to a model of type I diabetes in mice, two categories of transcripts predominate – those related to metabolic function and those related to inflammation.

The alterations in metabolic function and insulin signaling in diabetes are reflected in the endothelial transcriptome's response. Adipsin has previously been shown to be elevated in rats exposed to STZ-induced diabetes [11]. Adipsin and complement factor B assemble to enzymatically cleave complement factor C3 to C3a-des-arg/ASP (acylation stimulating protein) which stimulates triglyceride production in adipose tissue. Mice deficient in C3 exhibit resistance to diet-induced obesity [12]. We speculate that the endothelial up-regulation of adipsin reflects adaptive responses to fat utilization as the prime energy source in insulin-deficient animals. Apolipoprotein D is a potential carrier molecule for lipids and hormones [13]. Hsdl2 is potentially involved in intracellular lipid transport. Up-regulation of Slc36a2, an amino acid transporter [14], potentially reflects utilization of amino acids as alternative energy substrates. Our finding of increased adiponectin (a molecule that can enhance insulin signaling [15]) in our Type I model of diabetes contrasts with decreased adiponectin in human Type II diabetes[16].

Up-regulation of Cxcl2, a chemokine capable of inducing leukocyte rolling and extravasation in vivo [17] may contribute to immune-mediated vascular damage and the initiation of atherosclerotic lesions. We observed prominent up-regulation of Glycam 1, a glycoprotein expressed within the lymph node venous endothelium of mice[18]. Glycam1 can participate in lymphocyte attachment and extravasation into the blood vessel wall [19], and plasma levels of this molecule are found up-regulated in inflammation [20]. We observed increased Mcpt5, a mast cell protease considered to be a key regulator of angiotensin II expressed in cardiac endothelium [21]. Interestingly, Mcpt5 has been shown to be up-regulated in smooth muscle cells upon exposure to advanced glycation endproducts formed during diabetes [22]. Our observed increase in Gpx3 within the diabetic endothelium parallels increased Gpx3 within heart observed in a nearly identical model of streptozotocin-induced diabetes in mice [23]. Chi3l3 or -4, are members of a group of closely related, non-functional chitinases exhibiting increased levels during inflammation [24]. Ifi27, also known as Isg12, is a small intracellular protein induced by interferon signaling or other stress [25].

## Conclusion

Overall, our transcriptional analysis of the endothelium in an in vivo model of type I diabetes reveals the regulation of previously unsuspected genes. The products of these genes participate in basic responses of the vasculature to the combined effects of hyperglycemia, hypoinsulinemia, and inflammation associated with diabetes. These findings implicate pathways that are likely to be important in diabetic vascular pathology.

## Authors' contributions

JGM and RVS conceptualized the study. JGM planned and performed the experiments. JGM and RVS wrote the manuscript.
